# An Important Texas Work

**Published:** 1885-07

**Authors:** 


### AN IMPORTANT TEXAS WORK.

Dr. Geo. Cupples, of San Antonio, as chairman of a special committee on surgery, appointed by the State Medical Association to make a report of "all surgical operations performed in Texas," is now engaged in collecting detailed reports from the surgeons all over the State, and for that purpose will be pleased to forward to any whose names have been omitted, the necessary blanks and circulars of instructions. We are requested to say such reports should contain full details, such as "number of children in gynecological cases; chemical composition, and number of calculi in Lithotomy cases, subsequent health history, etc."

This work was began several years ago, by Dr. C., under the auspices of the State Association, and up to December 31, 1877, 1837 cases had been collected and recorded. These were comprised in thirteen classes, and his report, as far as completed, shows the ration of recoveries and deaths for each operation, and class of operation, and a general summary, giving the rates of recovery and death in major and minor operations, and in the sum total of operations; the number of cases of secondary hemorrhage, of erysipelas, of gangrene, of pyæmia, and of septicæmia, and, finally, the accidents resulting from the use of anæsthetics. Each one of the surgeons who have furnished reports, seventy in number, has been duly credited with each of his operations.

The committee will complete the work, so well begun, and to that end, surgeons are urged to communicate with the chairman.

This will be an exceedingly valuable and interesting work, and, when finished, we expect to see Texas surgery show up about as good results as that of any other State or country. It will set a praiseworthy example to other States, which, if followed, must result in a collection of statistics of inestimable value. This is doing for civil surgery what the collective investigation of disease is doing for medicine, and is just what the officers of the Bureau of the Surgeon General of the U. S. Army have done for military surgery in the magnificent Medical and Surgical History of the War between the States.

